# Rapid campimetry - a novel robust kinetic approach for visual field screening in glaucoma

**DOI:** 10.3389/fmed.2024.1419147

**Published:** 2024-08-02

**Authors:** Khaldoon O. Al-Nosairy, Katharina Rodenbeck, Sophie Vorholt, Nidele Djouoma, Hagen Thieme, Fabian Müller, Michael B. Hoffmann

**Affiliations:** ^1^Department of Ophthalmology, Faculty of Medicine, Otto-von-Guericke University, Magdeburg, Germany; ^2^Department of Optometry, Berlin University of Applied Sciences and Technology, Berlin, Germany; ^3^H & M Medical Solutions GmbH, Berlin, Germany; ^4^Center for Behavioral Brain Sciences, Magdeburg, Germany

**Keywords:** visual field, glaucoma, rapid campimetry, confounding VF factors, teleophthalmology

## Abstract

**Purpose:**

To investigate the robustness and variability of a novel kinetic visual field (VF) screening method termed rapid campimetry (RC).

**Methods:**

In RC visual field (VF) screening is enabled via kinetic-based testing on any computer (10°/4.7 s at 40-cm viewing distance) and high contrast in a dark room (1 cd/cm^2^). In experiment (1): 30 participants [20 healthy participants (HC), 5 glaucoma patients (GLA) and 5 patients with cataract (CAT)] were included to test the intra-session variability (fatigue effect) and the following effects on RC: room illumination (140 cd/m^2^), ±3 D refractive errors, media opacity. In experiment (2): Inter-session variability (1–3 weeks apart) was assessed in 10 HC and 10 GLA. Since RC detects absolute scotomas, the outcome measure was the size of physiological (blindspot) and pathological (glaucoma) scotomas in degrees. A repeated measures ANOVA was employed in experiment 1 and intraclass correlation (ICC) in experiment 2.

**Results:**

Neither the size of the blindspot nor the VF defects differed significantly between the different testing conditions. For intra-session variability, the average bias of blindspot size was −0.6 ± 2.5°, limits of agreement (LOA), in comparison to 0.3 ± 1.5° for VF defects, both with ICC of 0.86 and 0.93, respectively. For the inter-session repeatability, the average bias and LOA for blindspot size was 0.2 ± 3.85° in comparison 1.6 ± 3.1° for VF defects, both with ICC of 0.87 and 0.91, respectively.

**Conclusion:**

RC was robust to suboptimal testing VF conditions and showed good-to-excellent reliability between VF testing visits holding high potential for teleophthalmology.

## Introduction

Visual field (VF) testing is of great clinical importance for the assessment of diseases as in glaucoma, a global leading cause of irreversible blindness (1). However, current VF-testing methods, e.g., standard automated perimetry (SAP), endure several limitations such as long test duration, confounding factors and test-retest variability (2, 3). Recent advances in VF testing might hence provide alternatives that might be more robust to these changes. A kinetic VF test rapid campimetry (RC), was recently established as a fast, ≤ 1 min, method for VF screening in glaucoma specifically the central 10° VF (4). The importance of central VF testing in glaucoma even at very early stages of glaucoma, namely suspect glaucoma and ocular hypertension, is driven by evidence from functional and structural studies (5–8) confirming earlier established findings (8, 9) by Aulhorn (8) and Drance (9). According to Traynis et al. (10) “In any case, by using a 24-2 VF alone, clinicians can miss foveal and macular changes occurring before peripheral defects are present. Thus, it is important to use a VF test that better samples the central 10° than does the 24-2 test.” Moraes et al. (7) supports these observations and concluded “We suggest that clinicians should consider performing 10-2 tests not only in patients with established glaucoma but also in glaucoma suspects and ocular hypertensives to prevent misdiagnosis or misclassification of disease severity.” RC, by assessing the central VF, promises high potential in glaucoma given its short testing duration and interactive platform, and compatibility with tele-medical technologies. As RC thus holds the potential to be implemented anywhere in the world as a telemedical screening method, it is of great importance to assess its reliability and robustness in less controlled testing environments, e.g., home, and for suboptimal testing conditions such as uncorrected optical problems of the participants.

Generally, VF testing is limited by several confounds driving inaccurate identification of glaucoma diagnosis and progression. Proper lighting conditions, clear optical media, and proper refractive corrections are essential VF prerequisites that might influence test performance and results especially for the established threshold perimetries. These are factors that might be uncertain or even overlooked during, e.g., online testing. It is established that these factors influence standard VF tests and render testing unreliable (11–13). VF variability between visits or sessions can be another confounding factor, where VF sensitivities tend to fluctuate (14) and its reproducibility worsens with glaucoma progression (15). Thus the identification of glaucoma progression can be delayed which leads to the deterioration of visual outcome (16, 17).

The above confounding factors might challenge the potential of RC as a ubiquitous telemedical tool. In the present work, we, therefore, aimed to investigate RC robustness to the following confounding conditions, experiment 1: (i) ambient light, (ii) (simulated) cataract, (iii and iv) refractive error with +3 and −3 Diopters, (iv) fatigue (intra-session) effect. Finally, test-retest (intersession) reproducibility for 1–3 weeks’ tests was also assessed in a subset of participants in experiment 2. We hypothesized that RC is a sufficiently robust and valid VF method for inter-session testing.

## Methods and materials

This study is a prospective observational study conducted at the ophthalmology department, Otto-von-Guericke University (OVGU), Magdeburg, Germany, after ethical approval from OVGU local ethics committee (no. 151/16) following the tenets of Helsinki Declaration. Written informed consents were obtained from all study participants. Study endpoints are predefined based on the upon full recruitment of the required sample size.

### Participants

Experiment 1: 20 healthy participants [HC, 9 females (f), age: mean ± SD, 69 ± 4], 5 patients with mature cataract (CAT, 2f, age: 71 ± 9), and 5 glaucoma patients (GLA, 1f, age: 70 ± 10) were recruited in this experiment; the difference in age between groups was not statistically significant (ANVOA: *p* 0.924).

Experiment 2: 10 HC (5f, age: 68.7 ± 5.2) and 10 GLA (3f, age: 71.3 ± 6.4) participated in this experiment, the difference in age between groups was not statistically significant (*p* = 0.34).

All participants underwent complete ophthalmological examination including: (i) slit-lamp examination for anterior and posterior segments; (ii) measurement of best corrected visual acuity (BCVA) using early treatment diabetic retinopathy study (ETDRS) charts; (iii) visual field (VF) testing using the Humphrey Field Analyzer 3 (Carl Zeiss Meditec AG, Jena, Germany). One eye was selected per for measurement and analysis, see Table 1 for demographics. For HC, the eye was selected randomly; for GLA, the eye with more with VF damage was selected.

**TABLE 1:** Participants characteristics for experiment 1.

	HC (*n* = 20) mean ± SD	CAT (*n* = 5) mean ± SD	GLA (*n* = 5) mean ± SD	*P*-value	HC vs. CAT	HC vs. GLA	CAT vs. GLA
Age [Years]	69 ± 4	71 ± 9	70 ± 10	0.924	**–**	**–**	**–**
BCVA [logMAR]	−0.06 ± 0.11	0.28 ± 0.24	0.04 ± 0.11	**0.043**	0.07	0.192	0.265
SE [dpt]	(0.75, 4.5)	(0.0, 10)	(−0.25, 4.75)	0.087	**–**	**–**	**–**
**SAP**−**VF sensitivity**							
Fovea [dB]	36 ± 2	30 ± 3	35 ± 2	**<0.001**	**< 0.001**	0.850	0.**017**
VFI [%]	(99, 4)	(91, 13)	(49, 43)	**<0.001**	**0.003**	**<0.001**	**0.008**
MD [dB]	0.39 ± 0.90	−3.47 ± 3.17	−14.49 ± 5.25	**0.003**	0.109	**0.007**	**0.014**
PSD [dB]	1.50 ± 0.29	3.75 ± 2.66	13.66 ± 2.97	**<0.001**	0.255	**0.002**	**0.001**

HC, Healthy; CAT, cataract; GLA, glaucoma; BCVA, best corrected visual acuity in the logarithmic of minimal angle of resolution [logMAR]; SE, spherical equivalent; SAP, standard automated perimetry; VFI, visual field index; MD: mean deviation; PSD, pattern standard deviation; dB, decibels; dpt, diopter; (median, range). Bold *P-*values indicate significance.

Inclusion criteria for healthy participants were BCVA ≥ 0.8 and normal retinal exam and VF. For glaucoma, participants met the inclusion criteria for open-angle glaucoma with an open anterior chamber and typical glaucomatous optic disk damage defined by a vertical cup ratio ≥ 0.7, retinal nerve fiber layer defect or localized rim depression, and glaucomatous visual field defects with moderate to advanced glaucoma damage according to current guidelines (18).

Exclusion criteria were eye diseases, other than glaucoma or cataract in the respective groups, e.g., diabetic retinopathy, or systemic diseases, e.g., neurological diseases confounding visual function measurements. All participants except cataract group have no/or minimal age-related lens opacity that does not decrease BCVA < 0.8 decimal.

### Visual field testing

#### Standard automated perimetry (SAP)

Visual field was assessed in all participants using 24-2 SITA Fast protocol of the Humphrey Field Analyzer 3 (Carl Zeiss Meditec AG, Jena, Germany). For glaucoma participants, visual field defects were assessed further, for comparative purposes with RC, using 10-2 SITA Fast of the Humphrey Field Analyzer 3. All VFs are deemed reliable if met reliability indices of fixations loss and false negative rates < 33% and false positive rate < 20%.

#### Rapid campimetry

##### Test design and procedure

Rapid campimetry (4) is a kinetic-based test of central VF (17 × 10°) operated in any machine and run on a flat screen where a small light stimulus moves rapidly across the VF with a bright test dot (140 cd/m^2^) on a dark screen (0.8 cd/m^2^) at a standard viewing distance of 40 cm. The size of VF extended to 17° to ensure the detection of blindspot as a controlled fixation test. The test point changes its size as a function of distance from the fixation point, i.e., 1.05 mm (0.16°) near the fixation point and 2.72 mm (0.39°) in the blind spot region. The speed of test point was also optimized according to our previous observations where VF was tested with a running speed from 0.18 cm/s to 24 cm/s.

**FIGURE 1:**
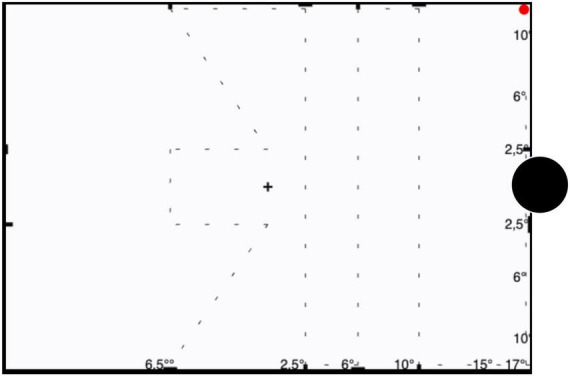
Illustration of trajectory of the test point. The test point trajectories are indicated for campimetry testing of the right eye as a dashed line (not shown during testing). The red circle denotes the start position of test spot which subsequently travels along the dashed line; the blindspot is indicated as a black disk not shown during testing. Participants are instructed to fixate the central cross during testing.

To accurately assess VF defects in glaucoma and following Aulhorn’s recommendations (8), the test point course, albeit arbitrary, was set to follow a trajectory that is related to the nerve fiber course as illustrated in Figure 1 (4), perpendicularly traversing arcuate scotoma as much as possible with seven paths covering a total length of ≈ 70 cm: (i) three vertical line courses temporally displaced from fovea to blind spot by 2.5°, 6°, 10° and 17°, (ii) two horizontal lines placed 2.5° from the fovea in either vertical directions running between fixation and a nasal displacement of 6.5° from fovea, and (iii) Two diagonal lines running from either vertical boundaries of VF starting nasally at 2.5° from fovea and ending in center. Along these paths, the test point moves vertically, diagonally, and horizontally through the VF and its size changes spontaneously depending on the distance from the fixation point. The area tested in rapid campimetry is a total 212.2 cm^2^ from the seven paths of the test point, and thus covers 6.75% of the paracentral visual field to be examined.

Patient sat in front of testing screen and the investigator monitored and marked the responses of the participant through a coupled observation screen. Before starting the test, each participant was familiarized with the test procedure by showing the how the point appears and disappears. This oriented participants to the testing procedure even if scotoma presents at the start location of the test point. Examiner was blind to the results of SAP.

As the test began at 17° outer/at the blind spot and the test-point ran automatically through the assigned paths, the subject signals the disappearance or reappearance of the test point. These points of the scotoma rim were marked by the investigator and the coordinates of these points are stored. Once a scotoma is detected, the investigator used a slower testing procedure to demark the scotoma boundaries which is important for reproducibility, an irrelevant procedure though for simple screening for scotoma presence. The examination output showed the two points (scotoma start and end) connected by a line symbolizing the presence scotoma at a given location. Identifying the scotoma boundary accurately was facilitated by reducing the running speed of the test point, e.g., by a factor of 4 or 8. Patients were allowed to blink spontaneously during examinations and to take breaks between testing conditions.

##### Experiment 1

Rapid campimetry testing of the assigned VF area took < 1 min per testing condition. To enhance fixation for this experiment, participants were asked to provide feedback during the test upon 1 s color change of the fixation cross every 5 s. Each experiment began and ended with standard VF testing conditions (T_*ST*1_ and T_*ST*2_) as detailed above under low room luminance. Four other confounds of VF tests were investigated, resulting in the below sequence:

(i) T_*ST*1_: Initial RC test in low luminance room 1 cd/m^2^
(ii) T_*Light*_: Testing with normal ambient room light, 140 cd/m^2^.
(iii) T_*Light*+*Filter*_: Testing as for T_*Light*_ viewed through blurring filters, Luminit filter (19), to simulate optic media opacities. The Luminit filter (1°) is an LSD filter (Light Shaping Diffuser), which is characterized by a uniform arrangement of microlenses and serves to simulate media turbidity. This resulted in a severe reduction of visual acuity to about 1.0 logMAR. According to Heinrich et al. (19), contrast sensitivity was reduced to such an extent that the test participants could hardly give any information about it and the corresponding value was then recorded as 0 logCS.
(iv) T_*Light*+3_: Testing as for T_*Light*_ viewed with a refractive error +3 dB.
(v) T_*Light–3*_: Testing as for T_*Light*_ viewed with a refractive error −3 dB.
(vi) T_*ST*2_: Second standard test, i.e., as for T_*ST*1_.

Due to influence of refraction on stimulus size for T_*Light*+3_ and T_*Light*–3_, a correction of the viewing distance was applied (20, 21) using the below formula:


$$Distance_{c\,o\,r\,r\,e\,c\,t\,e\,d}viewingdistance\left(40cm\right)/$$

$$\left(1-\frac{p\,o\,w\,e\,r_{c\,o\,r\,r\,e\,c\,t\,i\,n\,g\,g\,l\,a\,s\,s\,e\,s}}{m\,e\,a\,n\,r\,e\,f\,r\,a\,c\,t\,i\,v\,e\,i\,n\,d\,e\,x\,o\,f\,t\,h\,e\,e\,y\,e\,\left(66.73\,d\,p\,t\right)}\right).$$

##### Experiment 2

RC (condition T_*ST*_) was here repeated on two separate days (1–3 weeks) to investigate the intersession variability of RC-based VF screening and the experiment followed T_*ST*1_ standard procedure.

### Statistical analysis

After a normality check (Shapiro-wilk test), data were presented as mean ± SD or median and range for parametric and non-parametric data, respectively. For comparison between groups using parametric tests, the Welch ANOVA test was used if homogeneity of variance criteria was not met. A repeated measures ANOVA (RM-ANOVA) was applied to compare the size of blind spot measured by the rapid campimetry between healthy, cataract and glaucoma participants with one within-subject factor and one between-subject factors: (i) Test CONDITION, and (ii) GROUP, respectively. A one-way RM-ANOVA was run in glaucoma patients to compare the size of visual field defects to estimate the effect of test CONDITION factor. The mean size of the blindspot for each condition was normalized to T_*ST*1_:


$${\text{Relative mean size of blindspot = Size}}_{\text{ConditionT(i)}}/{\text{Size}}_{\text{T\_ST1}}$$

To estimate a potential intra-session variability, i.e., fatigue effect [condition 1 (T_*ST*1_) vs. 6 (T_*ST*2_)] and inter-session variability T_*ST*_ for 2 different days, a test-retest analysis was employed using Bland-Altman analyses and intraclass correlations (ICC). Reliability, based on ICC values, is interpreted as follows: Poor = ICC < 0.5; moderate = 0.50−0.75; good = 0.75−0.90; excellent = ICC > 0.90 (22).

Based on previous findings that SITA-FAST SAP tests and other visual field tests (VFs) (23, 24), such as the Melbourne VF, exhibit an intraclass correlation coefficient (ICC) of ≥ 0.9, a sample size of four participants is sufficient to reproduce these results. This calculation assumes an alpha level of 5% and a statistical power of 90%, as determined using the G*Power software (Heinrich-Heine-Universität Düsseldorf, Düsseldorf, Germany).^1^ *P*-values, if applicable, were corrected after Sidak, or Games-Howell (Welch ANOVA tests), for multiple comparisons/tests.

## Results

This study addressed the robustness of a novel method, rapid campimetry (RC), to screen VF defects in glaucoma. Potential confounding factors of VF measurements were investigated employing 2-way RM-ANOVA including the effects of 5 main CONDITIONS, i.e., T_*Light*_, T_*Light–filter*_, T_*Light*+3_, T_*Light*–3_, T_*ST*2_ on the size of physiological scotoma, blind spot, in three GROUPS, i.e., HC, CAT & GLA. We also probed the influence of such confounds on pathological scotoma (GLA) with a one-way RM-ANOVA on the mean size of the VF defects in GLA. Finally, a commonly established confound, intra/inter-session reproducibility, was assessed in a 2-step procedure: (i) intrasession variability by a comparison of the standard tests, T_*ST*1_ vs. T_*ST*2_; (ii) a follow-up experiment in a subset of participants, see methods, to analyze intersession variability.

### Confounding factors−physiological scotoma size

**FIGURE 2:**
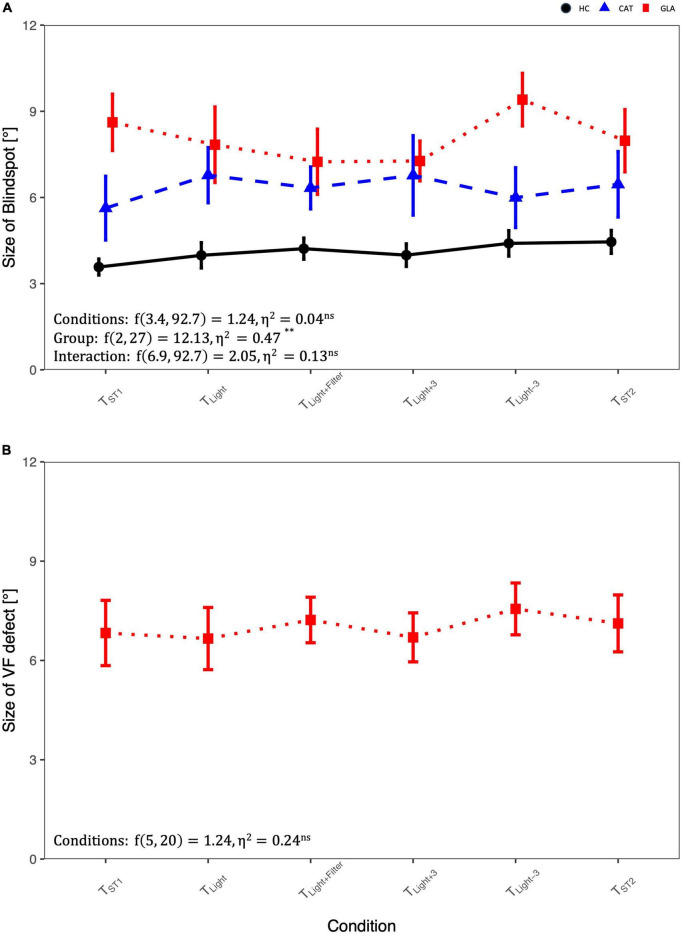
Comparison of blindspot and glaucoma-induced VF-defect size in different conditions using RM-ANOVA: **(A)** the size of blind spot differences across groups and conditions and **(B)** the size of visual field (VF) defect in glaucoma group. For abbreviations see Table 1.

Figure 2A and Table 2A show that the mean sizes of the blind spot for each group are comparable for different testing conditions. Table 2A lists also RM-ANOVA readouts where only the factor GROUP had, as expected, significant effects on the blind spots differences, which were due to the adjunct glaucomatous VF damage (GLA). Here the blind spot size in T_*ST*1_ was larger in GLA (8.6 ± 2.2) compared to HC (3.6° ± 1.3) and CAT (5.6° ± 2.5). Importantly, the main effect CONDITION, i.e., confound factors (see below), did not reach significance (*p* > 0.05). We performed a companion analysis on the normalized blind spot sizes. Table 2B presents the relative size of the blind spot calculated for each CONDITION relative to T_*ST*1_. Again, no significant effect of CONDITION was evident. This suggests that variable confounds did not affect the physiological scotoma size measured with RC.

**TABLE 2:** Analysis of blind spot size across different CONDITIONS and GROUPS.

A.	HC (*n* = 20)	CAT (*n* = 5)	GLA (*n* = 5)	Main effects		Interaction	*Post-hoc*		
	**m ± SD [°]**	**m ± SD [°]**	**m ± SD [°]**	**Test/condition f(3.4, 92.7) =**	**Group f(2, 27)=**	Test*Group f (6.9, 92.7) =	**HC vs. CAT**	**HC vs. GLA**	**CAT vs. GLA**
T_*ST*1_	3.6 ± 1.3	5.6 ± 2.5	8.6 ± 2.2	1.24, *p* = 0.30	12.13, *P* < 0.001	2.05, *p* = 0.06	0.06	** < 0.001**	**0.024**
T_*Light*_	4.0 ± 2.0	6.8 ± 2.2	7.8 ± 2.9				**0.047**	**0.004**	0.831
T_*Light–Filter*_	4.2 ± 1.7	6.3 ± 1.6	7.2 ± 2.5				0.079	**0.007**	0.818
T_*Light*+3_	4.0 ± 1.8	6.8 ± 3.1	7.3 ± 1.6				**0.029**	**0.008**	0.971
T_*Light–3*_	4.4 ± 2.0	6.0 ± 2.3	9.4 ± 2.1				0.346	** < 0.001**	**0.041**
T_*ST*2_	4.5 ± 1.8	6.5 ± 2.6	8.0 ± 2.4				0.164	**0.005**	0.572
**B. Relative size of blind spot to T_*ST*1_**				**Group comparisons**					
				***P*-value**					
T_*Light*_/ T_*ST*1_	1.1 ± 0.5	1.4 ± 0.6	0.9 ± 0.2	0.310					
T_*Light–Filter/*_ T_*ST*1_	1.2 ± 0.5	1.3 ± 0.6	0.8 ± 0.1	0.225					
T_*Light*+3/_ T_*ST*1_	1.1 ± 0.5	1.4 ± 0.8	0.9 ± 0.2	0.357					
T_*Light–3/*_ T_*ST*1_	1.3 ± 0.6	1.3 ± 0.9	1.2 ± 0.6	0.960					
T_*ST*2/_ T_*ST*1_	1.2 ± 0.3	1.3 ± 0.7	0.9 ± 0.2	0.233					

HC, Healthy; CAT, cataract; GLA, glaucoma. Bold *P-*values indicate significance.

### Confounding factors−pathological scotoma size

Figure 2B depicts the influence of the potential confounds upon the mean size of pathological scotomas, i.e., glaucomatous VF defects. As reported above for the blind spot, there was no significant main effect of CONDITION on the size of VF defects, *p* > 0.05.

### Intra- and intersession reproducibility

**FIGURE 3:**
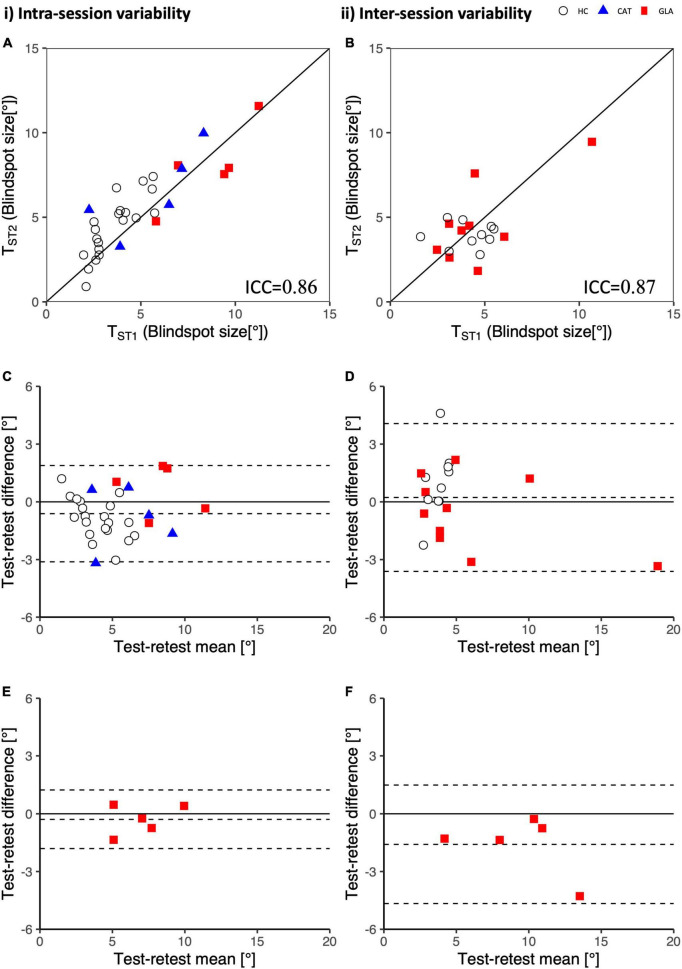
Intrasession and intersession variability of the standard rapid campimetry tests (T_*ST*_): Subplot (i) on the same day and subplot (ii) on different days, 1–3 weeks apart. **(A,B)** Correlation plots for blindspot’s size. Bland-Altman plots for **(C,D)** blindspot’s size reproducibility and **(E,F)** VF defects’ size reproducibility.

The intra- and intersession reproducibility was assessed via inter-session correlation and Bland-Altman analyses, as depicted in Figure 3. For intra-session variability, T_*ST*1_ and T_*ST*2_ for physiological [pathological] scotomas had good [excellent] reliability as shown by ICC = 0.86 [0.93] (95% CI of ICC: 0.73 to 0.93 [0.51 to 0.99]). The average bias calculated via a Bland-Altman analysis was −0.6° ± 2.5° [0.3° ± 1.5°]. It should be noted that the variability, as quantified by 95% limits of agreements (LOA), −3.1 to 1.9° [−1.9 to 1.2°], indicates the 95% CI of the data.

The intersession variability showed a comparable concordance, ICC = 0.87 [0.91], (95% CI of ICC: 0.71to 0.95 [0.35 to 0.99]) between visits. Intersession variability showed a bias of 0.2° ± 3.85° [−1.6° ± 3.1°] with 95% LOA of −3.6 to 4.1° [−4.7° to 1.5].

## Discussion

Visual field testing remains one of the most common tests performed in glaucoma management but its lack of ubiquity, multiple confounding variables, and the test-retest variability of current standard methods, SAP, challenge its potential in glaucoma diagnostics in general and its particular use for cloud and telemedicine technologies. Rapid campimetry (RC), a kinetic-based method, was highly sensitive to screen for VF defects in surprisingly short time, < 1 min (4). In this study, we probed RC validity and robustness towards common confounding factors and its test-retest variability. RC showed robustness to ambient light, blurring by refraction error, and media opacity which might promise its utility as an effective telemetric VF screening method not only in clinically operated units but even at homes upon further optimizations. High intra/inter session reproducibility, ICC > 0.85%, supported these findings.

In our previous work (4), we demonstrated that RC was comparable to SAP in the detection of absolute scotomas and actually outperformed the standard method in the detection of glaucomatous arcuate scotomas. Motion-based perimetry holds high potential in glaucoma diagnostics even at the earliest stages (25, 26). At more advanced glaucoma stages, test-retest variability is a limiting factor of static perimetric tests (27). A recent study (28) investigating a new motion-based perimetry in glaucoma, supported the notion that motion based perimetry is not only favored by participants, but also demonstrated lower test-retest variability (−6.35 to +6.48 dB vs. −12.7 to +7.81 dB) in comparison to static stimuli. Low test-retest variability of moving stimuli might be due increasing sensitivity of detectability due to the stimulation of motion-sensitive retinal ganglion cells (29) besides stimulation of motion-driven middle temporal neurons (30) in comparison to sole light stimulation in static perimetry.

As RC only requires a standard computing set-up, it holds potential for cloud technologies and online testing. Consequently, the present study aimed to test its robustness to home-based or suboptimal environments. In this context, RC was robust towards ambient room light which might related to the used suprathreshold stimulus. The performance of the Melbourne VF perimeter, a static portable perimeter, was not influenced by the ambient illumination but authors recommend testing in a dimly lit room, as for the Humphrey field analyzer (HFA), to maintain largest possible dynamic range of stimulation (31). In fact, robustness to ambient light might simulate doing tasks in real life and facilitate testing under different daily conditions. Another factor that furthers the use of RC as a screening tool for VF defects is its robustness to (simulated) cataract. Cataract [or simulated (13)] is known to severely influences conventional VF testing (11, 32) where it might even mask VF changes in glaucoma (33, 34). Further, it was found that cataract might overestimate pattern deviation damage in glaucoma which warrants ophthalmologists attention (35). Melbourne VF testing, employing threshold stimulation, did find a significant effect of blurring on reduction of VF sensitivity (31). Again, the suprathreshold stimulation allows for such robustness of RC even against refractive errors mimicking situations at home without proper refraction/glasses. In conventional VF methods, however, incorrect refraction might mimic isolated central VF defects and each 1 diopter of uncorrected refractive error might lead to 1.26 dB loss of retinal sensitivity (12, 36). The importance of refractive correction is also important even for peripheral VF testing (37).

### Current limitations, clinical applications and future perspectives

Rapid campimetry is found to be robust against most common confounds in examinations routine of VF, which highlights its promise as a screening test and potential, upon proper future optimizations, namely to be fully automated rather than, as at present, operator dependent, for telemedical technologies. This involves optimizations of the testing procedure, e.g., to be fully automated and operated independently by the participant. Monitoring eye fixations with RC testing, not performed and hence another limitation in the present study, will establish one important reliability parameter in VF testing, i.e., fixation loss. Study participants were reminded to keep fixating at the central cross during the RC procedure to avoid any fixation loss. For experiment1, patients were engaged in a fixation target task (report brief color change (1 s) fixation cross every 5 s), thus encouraging fixation. This should be further addressed along with other reliability indices, false positives/negatives, using larger sample size, e.g., a multicentric study, which is of importance to establish RC as a complementary tool for VF examinations. Further, optimization of testing paradigm where a participant could conduct himself independent of any operators, is another future endeavor.

Rapid campimetry is designed to screen the central 10° of VF, an important region in glaucoma examinations. Established evidence consolidated the importance of central 10° not only in advanced stages but also in early stages of glaucoma even in glaucoma suspects (5–8). Besides, central 10° of VF is significantly associated with the quality of life of affected individuals (7, 38, 39). However, current testing procedure, e.g., 10-2 SAP, might be hampered by limitations including the lack of patient-compliance and long testing times. RC can overcome these limitations with fast recordings and being interactive. For an additional comparability to standard 24-2 perimetry, RC is currently being developed for testing of a larger visual field, i.e., 30°. This will meet needs to test peripheral VF affected usually at the start of disease process in glaucoma.

## Conclusion

This study provides evidence that rapid campimetry is a robust and reproducible VF-testing method even in patients with advanced glaucomatous visual field defects and optic media opacities. This motivates a larger sample size multicentric study for further validation in order to establish the rapid campimetry as a screening tool for VF examination with potential for telemedical technologies.

## Data availability statement

The raw data supporting the conclusions of this article will be made available by the authors, without undue reservation.

## Ethics statement

The studies involving humans were approved by the Otto-von-Guericke University local ethical committee (no. 151/16). The studies were conducted in accordance with the local legislation and institutional requirements. The participants provided their written informed consent to participate in this study.

## Author contributions

KA-N: Conceptualization, Data curation, Formal analysis, Investigation, Methodology, Supervision, Validation, Visualization, Writing−original draft, Writing−review and editing. KR: Data curation, Formal analysis, Investigation, Writing−review and editing. SV: Data curation, Formal analysis, Investigation, Writing−review and editing. ND: Writing−review and editing. HT: Resources, Writing−review and editing. FM: Formal analysis, Software, Writing−review and editing. MBH: Conceptualization, Funding acquisition, Project administration, Resources, Supervision, Writing−review and editing.

## Footnotes

1
http://www.gpower.hhu.de/
